# Gender and autistic traits modulate implicit motor synchrony

**DOI:** 10.1371/journal.pone.0184083

**Published:** 2017-09-05

**Authors:** Miao Cheng, Masaharu Kato, Chia-huei Tseng

**Affiliations:** 1 Department of Psychology, University of Hong Kong, Hong Kong SAR, China; 2 NTT Communication Science Laboratories, NTT Corporation, Atsugi, Japan; 3 Center for Baby Science, Doshisha University, Kyoto, Japan; 4 Research Institute of Electrical Communication, Tohoku University, Sendai, Japan; Leiden University, NETHERLANDS

## Abstract

Interpersonal motor synchrony during walking or dancing is universally observed across cultures, and this joint movement was modulated by physical and social parameters. However, human interactions are greatly shaped by our unique traits, and self-related factors are surprisingly little studied in the context of interpersonal motor synchrony. In this study, we investigated two such factors known to be highly associated with motor coordination: gender and autistic traits. We employed a real-world task extending our understanding beyond laboratory tasks. Participants of the same gender were paired up to walk and chat in a natural environment. A cover story was introduced so that participants would not know their walking steps were being recorded and instead believed that their location was being tracked by a global positioning system (GPS), so they would ignore the motor recording. We found that the female pairs’ steps were more synchronized than those of the males, and higher autistic tendencies (measured by the autism-spectrum quotient) attenuated synchronous steps. Those who synchronized better had higher impression rating increase for their walking partners (measured by interpersonal judgement scale) than those who synchronized less well. Our results indicated that the participants’ joint movements were shaped by predisposed traits and might share similar mechanism with social functions such as empathy.

## Introduction

Interpersonal motor synchrony is commonly observed in daily life. One kind of interpersonal motor synchrony is explicit and intentional, which usually occurs in a joint action requiring individuals deliberately moving along each other, for example, dancers coordinated body movements with their partners or soldiers sync steps in a military parade. The other kind, the focus of current study, is implicit and spontaneous, such as when individuals unintentionally sync their walking steps with others or audience automatically clapping[1] in a synchronized pattern. Although both kinds of synchrony involve simultaneous actions, explicit synchrony is both the purpose and the result of motor coordination while implicit synchrony is not the purpose but the result of motor coordination.

Recently, interpersonal synchrony and its confining factors such as physical parameters have attracted much research attention. A walking dyad with similar leg lengths tended to have more synchronized steps[2], and people exhibited less synchrony while rocking in chairs of vastly different weights[3]. When waving swords or clapping hands, dyad partners with differential weights attached to their wrists were less synchronized with each other[4]. When swinging pendulums, pairs holding pendulums of similar length were more stably and synchronously coordinated[5]. The interpersonal motor synchrony affected by discrepancies of a dyad’s physical characteristics, from the perspective of coordination dynamics, is similar to the entrainment of two metronomes on a shared board affected by their natural frequencies. Smaller differences between two agents’ intrinsic rhythms create less phase lag and promote the rhythmic synchronization of inanimate oscillations as well as movement harmony within a dyad[4]. Among these studies, participants’ movements were tracked by motion sensors or motion capture markers placed on the participants’ joints[2,4], the headrest of rocking chairs[3], and the end of pendulum[5]. In most cases, participants were requested to coordinate their movements with their partners and were aware of their movement being recorded.

Social factors related to others such as impressions and social relations also modulate the coherence and stability of interpersonal motor synchrony. For example, inherent and primed pro-social attitudes promote synchronous arm curls[6]. People tend to show more stable motor synchrony with a partner who is perceived more attractive[7] and punctual[8]. Besides, greater disparities in social competence[9] and aesthetic preferences[10] between two agents evoke greater interpersonal synchrony. Paxton and Dale [11] discovered that arguments inhibited the interpersonal convergence of body movements compared to less competitive conversations, while Tschacher et al.[12] found that a debate promoted greater levels of body synchrony than a cooperative conversation. Most of above-mentioned studies[7–10] required participants to coordinate their movements with an assigned partner while the motion sensors were attached to the participants’ joints, and participants possibly became aware of and placed attention on their motion synchrony. Two studies[11,12] were able to tap it in a more implicit way by misleading participants to think that the research aimed for conversation study, and participants’ body motion recorded by hidden cameras was analyzed afterwards.

Another similar phenomenon that also involves interpersonal nonverbal behavior matching, non-conscious mimicry (i.e. spontaneous or automatic imitation), has been extensively studied. Non-conscious mimicry refers to spontaneous imitation of body movement (e.g. face rubbing and foot shaking) and facial expression[13], and it is also closely interacts with social factors[14,15]. Automatic mimicry behavior evokes positive social consequences [14,15], which includes increased liking[13], rapport[16], prosocial behavior[17,18], and reduced radical prejudice[19]. Social factors, such as in-group membership[20] and romantic interest for an attractive stranger[21] also boosted automatic mimicry frequency. Furthermore, individual differences predispose one’s tendency to mimic other’s behavior[15]. For example, people with secure attachment style tend to exhibit more mimicry behavior than people with insecure attachment style[22]. However, little is known whether implicit motor synchrony is also constrained by individual differences.

While influences from the social relation with others have been studied, fewer studies have investigated whether self-related factors may also predispose individuals to processes of interpersonal synchronization in predictable ways. In this study, we fill in the research gap by investigating the impact from two factors on motor synchronization.

The first factor is gender, which was a well-acknowledged factor to modulate our motor performance. Males have better gross motor performance than females in various activities (e.g., cutting maneuvers, treadmill locomotion, throwing, ball skills, and object control proficiency) from childhood[23–25] to adulthood[26–28], possibly because males are more physically active since the beginning in childhood[29–31]. However, do men’s motor advantages stand when it comes to coordinating with others? In early infancy, mother-son pairs are more likely than mother-daughter pairs to sync their gazes and affective behaviors during face-to-face interactions[32,33]. In a recent study, Valdesolo, Ouyang, and Desteno found that synchronizing in rocking chairs enhanced performance in a subsequent joint motor coordination task[34]. Although little evidence from adult studies reports gender difference in interpersonal motor synchrony, it is tempting to speculate that males may benefit from their motor coordination abilities and sync better because both motor coordination activities and interpersonal synchrony require skills of analyzing anticipating external feedback and act accordingly at a precise timing[35].

However, as motor synchrony is also modulated by social factors with others, in which females are known to be superior (e.g., empathy and social communication), it leads to an opposite prediction: females sync better than males. It has been well studied that females’ strength in decoding non-verbal cues. Perceptually, females are more sensitive than males to non-verbal cues such as emotion in faces[36–41] and biological motion[42,43]. Females also have stronger brain activity in areas related to social perception while processing biological motion[44]. In addition, females are also more emotionally expressive than male in facial expressions[41,45,46] and voice expressions[47]. Beginning in early infancy, females are more self-regulating in emotion expression; specifically, they show more positive and fewer negative emotions than males[33,48]. Last but not least, females feel more emotional contagion and mimic facial expression more than males[41]. Therefore, it is reasonable to hypothesize that that females are also more responsive than men to other’s body movements, especially in terms of synchronizing their bodies to others’. It’s intriguing to see how the two opposite predictions are reconciled in empirical studies and to investigate which factor contributes more to modulating implicit motor synchrony: motor abilities or social skills.

The second factor of our interest is autistic traits, which was motivated by the growing number of reports in clinically diagnosed autistic spectrum disorders (ASD) individuals. ASD patients, in addition to the well-known motor deficits[49–53], also show less interpersonal synchronization when required to intentionally take action in time with others[54], implicitly sync with caregivers in rocking chairs[55,56], and they also perform worse in imitation tasks[57–59] and social motor coordination tasks[54,60]. ASD participants have lower interpersonal brain synchrony in social brain areas when watching a film presenting social interactions compared to the typically developed (TD) population[61]. There is only one report that we are aware of that suggested that autistic traits were associated with interpersonal motor synchronization in healthy individuals. Schmidt and colleagues[62] revealed that less bodily synchronization was observed in a joke telling task when the responders were more autistic measured by autism-spectrum quotient (AQ) questionnaire[63]. However, individuals with high autistic tendencies are also known to have trouble understanding jokes[64], and motor synchrony is also influenced by agreement levels within a verbal communication[11]. It is possible that other cognitive factors such as incomprehension of jokes in addition to autistic tendency also contributed to reduced motor synchrony. As autistic trait is believed to be a continuum among the non-clinical population[63], it will add to the accumulated knowledge in the field by investigating whether the healthy individuals’ autistic traits also connect to their behavioral synchrony.

The current study investigates healthy participants’ gender, permanent traits (especially, autistic tendencies), and implicit interpersonal motor synchronization in a natural, everyday situation. Participants, meeting for the first time, walked along a pleasant path while freely talking about any topics of their choice. Normally, such an ice-breaking conversation includes contents that both parties fully comprehend, so that we can avoid possible confounding from incomprehension of communication such as in a joke-telling task[62]. Participants’ steps were recorded with motion sensors disguised as GPS to preclude their awareness of the motion recording so they would not pay excessive attention to their movement.

## Methods

This study applied between-subject design and we intended to recruit 60–80 pairs in each gender based on a study done by one of our co-authors [65]. For data collection efficiency, we hoped to pair each participant with every other one to maximize the possible number of pairs. Due to motor sensor availability, we planned to recruit eight participants in each session. The final number varied from 4–8 due to the enrollment situation (e.g. no-show).

### Participants

A total of 31 female and 27 male participants were recruited. In all we conducted five sessions with females and four sessions with males. All participants provided written informed consent to participate the experiment. They were told a cover story, and the real purpose was disclosed to them at the end. All the procedures were approved by the Human Research Ethics Committee of the University of Hong Kong (Reference No. EA231012). All methods were performed in accordance with the relevant guidelines and regulations.

### Procedure

After participants arrived at the meeting room, they were instructed to fill out a personal information form (including age, height, weight, and foot length) and autism-spectrum quotient (AQ) questionnaire[63] (see S1 File for Chinese translation). Participants also rated their first impressions of every other participant using an interpersonal judgment scale (IJS)[66] (see S2 File for Chinese translation) (Byrne, 1971). The experimenter gave the participants misleading instructions to make them believe that this study was about how communication content affected interpersonal liking, and that the experiment would be conducted while walking to simulate a natural situation for communication and avoid interference from others. The walking path was part of a quiet and barrier-free path inside the University of Hong Kong. The distance was fixed, about 350 m, and it took participants approximately 6–9 minutes to finish one round trip. After filling out the AQ questionnaire and the pre-experiment IJS on their first impressions of the other subjects, participants walked individually to get familiar with the walking path. They were then paired with each other participant, successively, and immediately completed a post-experiment IJS, rating each partner again.

During the walks, the participants wore voice recorders. Their walking movements were recorded by acceleration sensors (ATR-promotions, TSND121) disguised as a GPS device, which were attached above their right ankles. The session continued until each participant had walked with every other participant in that session. Usually one session lasted 1–2.5 hours.

## Data processing

The data processing details are shown in supplementary materials. We extracted the acceleration data from motion sensors and used auto correlation to obtain participants’ duration required to complete one step (i.e. pitch), an indicator for walking tempo (our sensor did not provide walking distance, so walking speed was not available). Cross-correlation is to compute the lag between two walkers’ footsteps, and the total duration when two walkers were phase-synchronized relative to the total walking period was defined as the phase synchronization time ratio[65].

## Results

A total of 31 female and 27 male participants formed 84 female pairs and 81 male pairs. Because previous study showed that motor synchrony was affected by social relation[10,65], we only included pairs of strangers who had never met each other before. After excluding 12 pairs who were acquaintance prior to the experiment, the data analysis was based on 77 female pairs and 76 male pairs. Age-related data analysis only had a sample size of 71 female pairs and 76 male pairs because of data loss. Female pairs were significantly younger (27.82 vs. 30.58 years old, *t*(145) = 2.19, *p* = .030), shorter (161.59 cm vs. 173.99 cm, *t*(151) = 19.63, *p* < .001), lighter (53.26 vs. 64.50 kg, *t*(151) = 15.91, *p* < .001), and had lower AQ (17.45 vs. 20.33, *t*(151) = 5.16, *p* < .001).

### Gender, AQ, and walking tempo (pitch)

**Fig 1:**
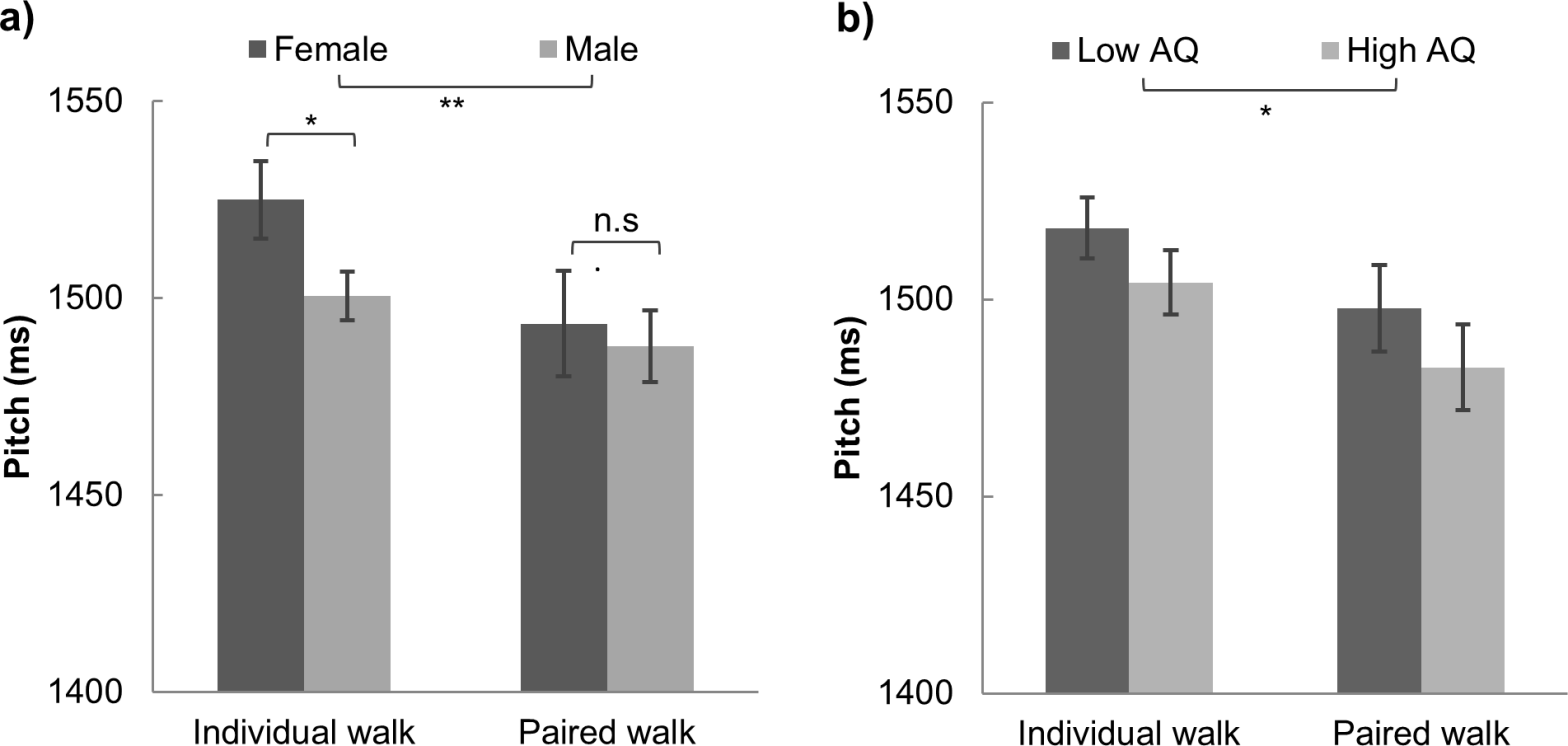
Gender, AQ and participants’ walking pitch. Means and 95% confidence interval for walking pitch (duration for a complete footstep) for a) female and male dyads and b) low and high AQ pairs.

We first examined whether walking tempo associates with personal traits by applying a two (gender, between-subject) by two (individual/paired walk, within-subject) mixed designed ANOVA on the duration required per step (i.e. walking pitch, Fig 1A). We found significant interaction effect between two factors, F(1, 256) = 6.293, p = .013, η^2^ = .024. Post-hoc tests revealed that the gender difference occurred only in individual walk (t(256) = 4.349, p < .001), but not in paired walk (Female = 1489.8, Male = 1487.7, p = .780). A similar 2 by 2 ANOVA was applied on walkers’ AQ and the walking conditions (Fig 1B). Low AQ walkers had greater pitch than high AQ ones (F(1, 256) = 6354, p = .012, η^2^ = .024). Similarly, people walk with significantly higher pitches in individual walk (F(1, 256) = 30.925, p < .001, η^2^ = .108). The interaction effect was not significant, p = .875.

### Gender, AQ, and walking synchrony

**Fig 2:**
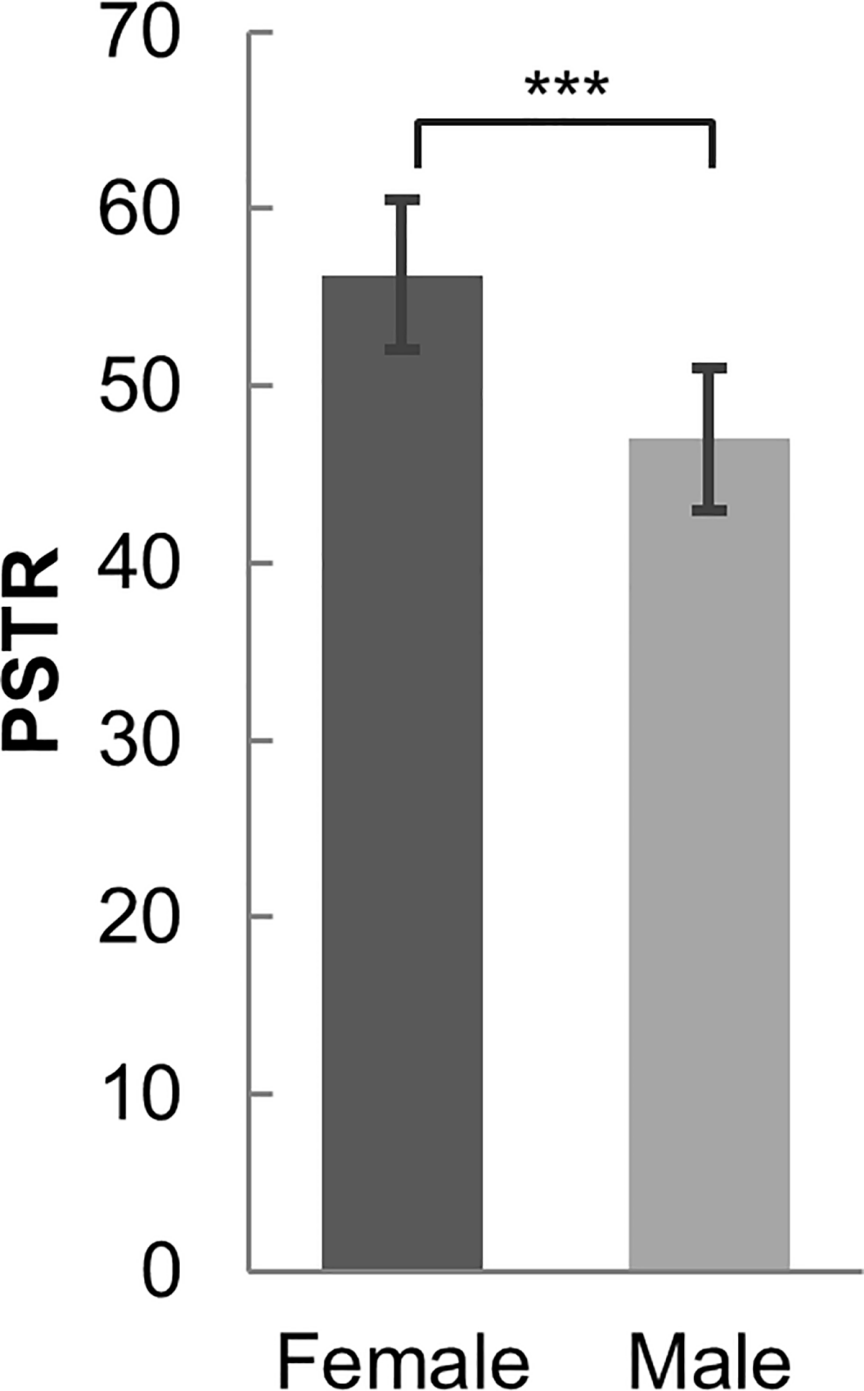
Gender difference in walking synchrony. Means and 95% confidence intervals for phase synchronization time ratio (PSTR) of female and male dyads (***, *p* < .001).

To estimate the gender difference of walking synchrony, a one-way between-subject analysis of covariance (ANCOVA) was conducted on phase synchronization time ratio (PSTR), controlling for the AQ mean, age mean, height difference, and weight difference of each dyad. Because participants’ height and feet size were significantly correlated (*r* = .816, *n* = 57, *p* < .001), feet size is not included for analysis to avoid redundancy. Female pairs had significantly higher PSTR (55.09) than male pairs (48.08), *F* (1, 141) = 8.246, *p* = .005, *η*^*2*^ = .055 (Fig 2). The covariate, AQ mean of a dyad, was significantly related to PSTR (*F* (1,141) = 4.928, *p* = .028, *η*^*2*^ = .034).

**Fig 3:**
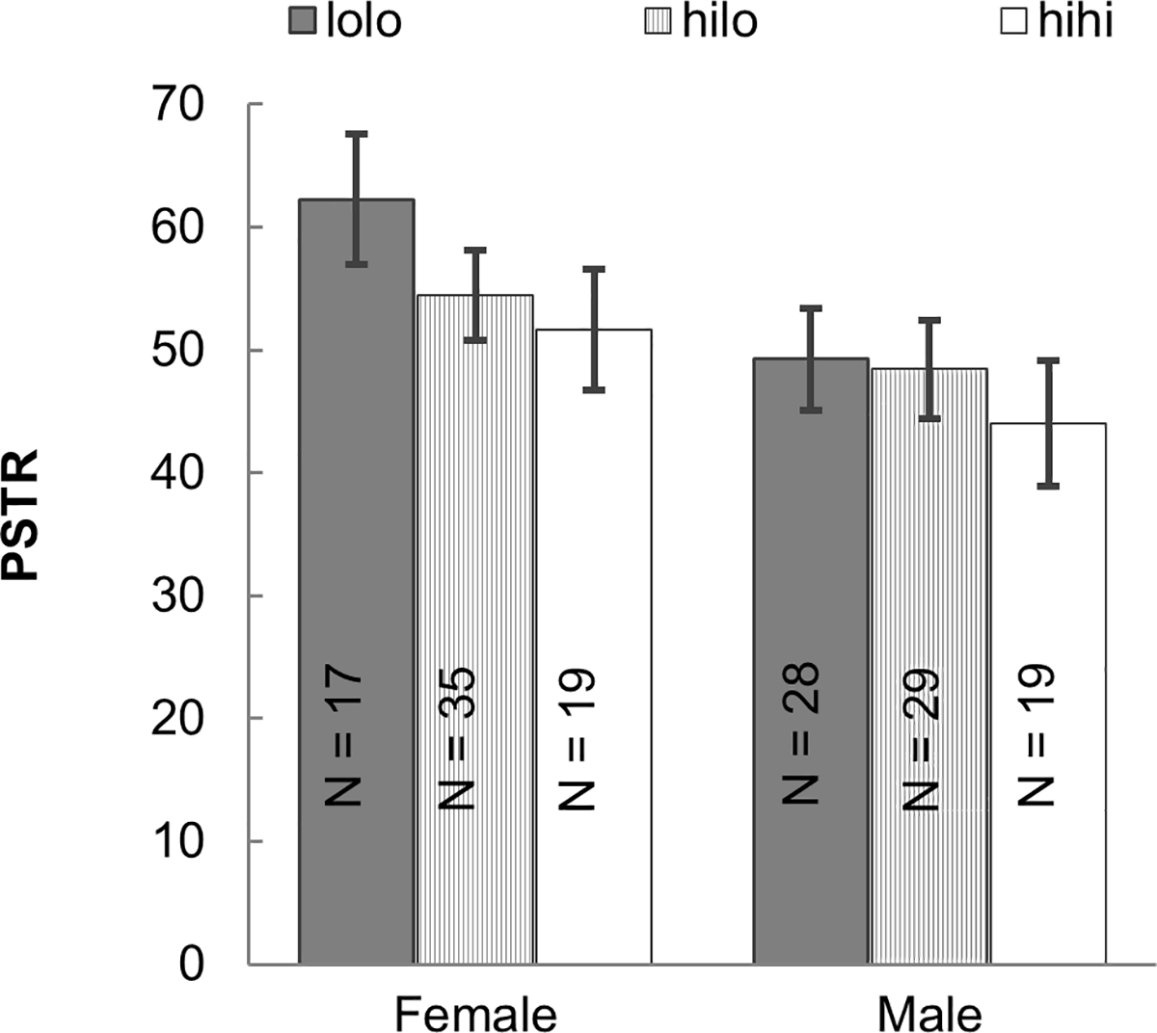
Walking synchrony in different AQ groups of each gender. Means and 95% confidence intervals for PSTR of female and male pairs in different AQ combination groups: high AQ with high AQ participant (hihi), high AQ with low AQ participant (hilo), and low AQ with low AQ participant (lolo). The AQ average of each gender group was used to classify all participants as either high or low AQ.

To understand how each individual’s AQ influenced walking synchrony, we classified all participants as either high or low AQ individuals with the average AQ (F: 17.45, M: 20.33). All pairs belonged to one of the three possibilities: low AQ with low AQ (lolo), high AQ with low AQ (hilo), and high AQ with high AQ (hihi). A two-way between-subject ANCOVA considering gender and AQ groups (lolo, hilo, and hihi) was conducted on PSTR, controlling for the age mean, height difference, and weight difference of the pairs. The main effect of AQ group, *F*(2, 138) = 4.977, *p* = .008, *η*^*2*^ = .067 was significant, as was gender, *F*(1, 138) = 20.44, *p* < .001, *η*^*2*^ = .129. Planned contrasts revealed that lolo pairs (PSTR mean = 55.74 56.50) synced better than hihi pairs (hihi group: PSTR mean = 47.85, *p* = .002). The interaction between gender and AQ group was not significant, *p* = .253 (Fig 3).

Furthermore, we applied a multiple linear regression model to investigate which AQ subscales had the greatest effect on body coordination. Five AQ subscales (*social skills*, *attentional switching*, *attention to details*, *communication skills*, and *imagination*) were used to predict PSTR. The model explained a significant amount of variance in PSTR: *F*(5, 147) = 4.734, *R*^*2*^ = .139, *p* < .001. Attentional switching (*Beta* = -.195, *p* = .028) and imagination (*Beta* = -.240, *p* = .005) significantly contributed to predict PSTR. The results imply that participants who received low scores in attentional switching and imagination synchronized less in walking than those who scored high.

### Gender, AQ, and tempo adjustment during paired walking

Because footsteps tempo adjustment is required when two people walk side by side, we examined whether the flexibility of adjustment relates to one’s gender or AQ. For every walker, the tempo adjustment was defined as the difference between pitches in paired walk and in individual walk. The final analysis included 129 pairs after excluding 18 pairs data loss and additional 6 pairs with equal AQ scores.

**Fig 4:**
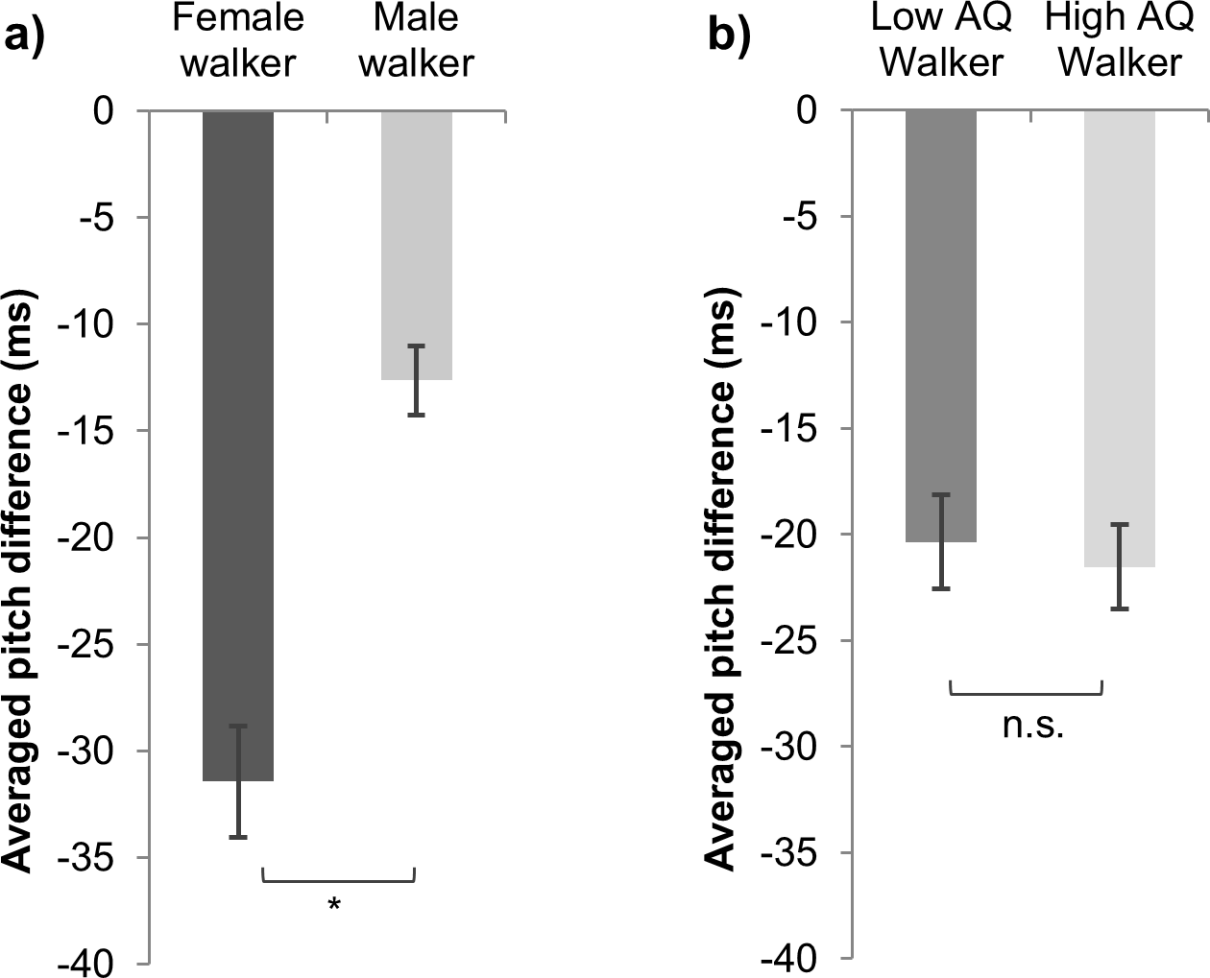
Gender, AQ, and tempo adjustment. Tempo adjustment (i.e. the difference of an individual’s pitch when s/he walks alone and when s/he walks with a partner (pitch_paired walk_−pitch_individual walk_) means and 95% confidence intervals between a) walkers of two genders, and of b) different AQ groups (*, *p* < .05).

We found significantly more drastic tempo adjustment in female walkers (31.443) than male ones (-12.642, *t*(256) = 2.509, *p* = .013), which implied that female walkers were more likely to adjust their walking tempo to their partners (Fig 4A). Tempo adjustment from high AQ group (average = -4.308) is not significantly different from the low AQ group (average = -4.072, *t*(256) = 0.157, *p* = .875), which suggests that autistic trait didn’t modulate how much one would adjust oneself to other’s walking tempo (Fig 4B).

### Walking synchronization and social consequence

**Fig 5:**
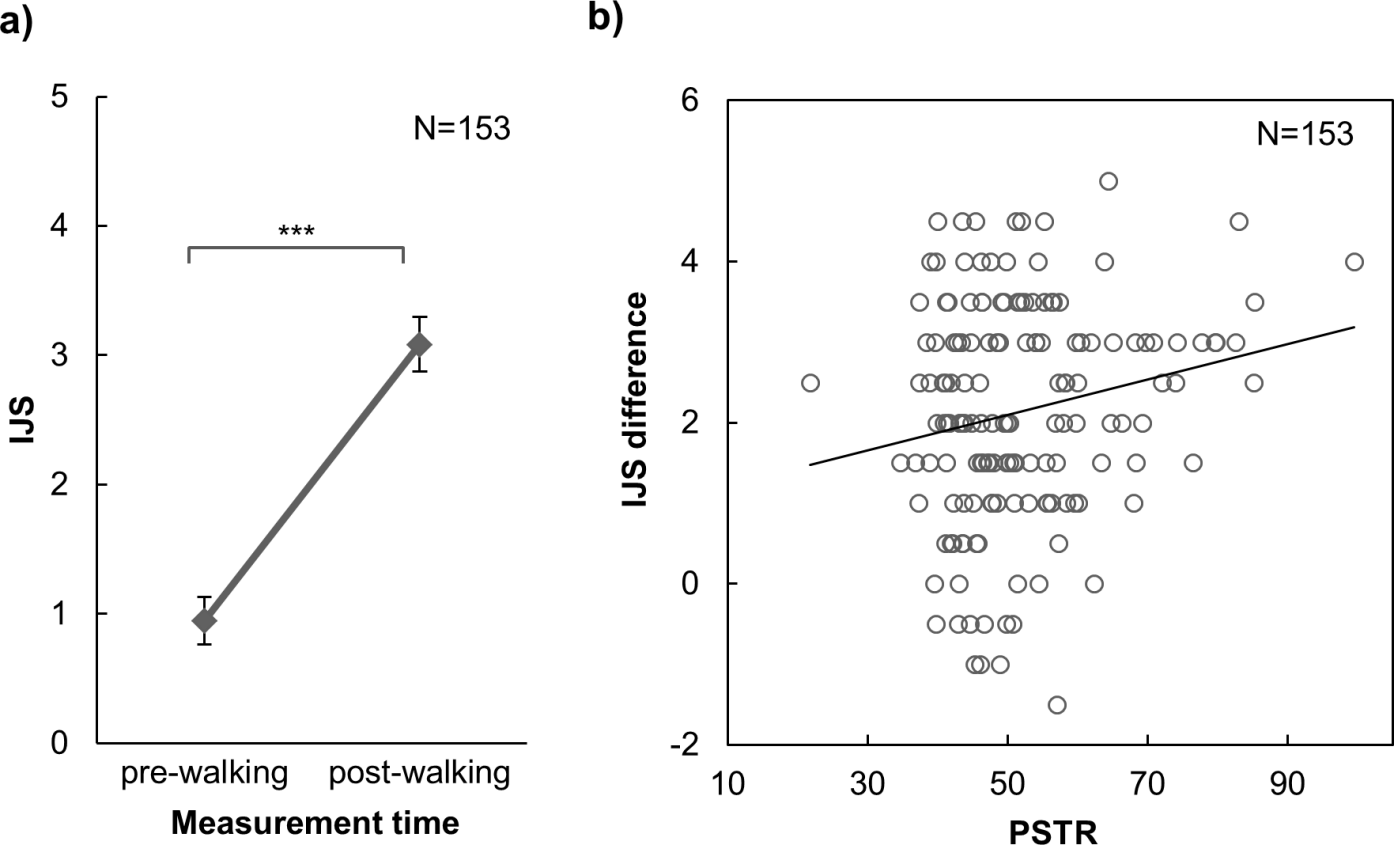
Results of walking synchrony and impression. a) Means and 95% confidence intervals for two walkers’ total pre- and post-IJS scores. b) IJS difference and PSTR of each pair. IJS difference was obtained by subtracting the mean of two walkers’ pre-IJS scores from the mean of their post-IJS scores (***, *p* < .001).

In general, participants had better impressions of their partners after walking and chatting (Fig 5A). As evidence, a paired *t*-test showed that two walkers’ average rating sores in interpersonal judgment scale (IJS) obtained after they were paired to walk (3.085) were significantly higher than the average pre-IJS scores (0.948), *t*(152) = 19.185, *p* < .001. In particular, female dyads’ positive interpersonal impressions were significantly more enhanced than those of the male dyads (*t*(151) = 2.312, *p* = .022, 95% CI[0.148, 1.884]). The improvements in participants’ impression changes during a paired walk was positively correlated with their motor synchrony with their partners (*r* = .190, *n* = 153, *p* = .019, Fig 5B), which indicated that better-synced pairs showed greater increases in interpersonal impressions.

Because gender and AQ modulate PSTR, the correlation between PSTR and IJS differences might actually be affected by gender and AQ. We examined the association between PSTR and IJS while controlling gender and AQ. With respect to AQ, a significant partial correlation (*r*(150) = .167, *p* = .040) between PSTR and IJS difference was still observed. After gender effect was controlled, we found a marginally significant partial correlation (*r*(150) = .140, *p* = .086) between PSTR and IJS difference, implying that the effect was weaker than previously thought. To further explore the gender effect, we correlated PSTR with IJS differences separately for each gender. Female dyads showed a marginal positive correlation (*r*(77) = .193, *p* = .092), which suggested a promising interplay between walking synchrony and affection in female dyads. Male dyads showed no tendency of correlation(*r*(76) = .075, *p* = .521) between PSTR and IJS.

### Conversation content

Although we did not conduct a systematic analysis on the conversation contents due to resource limitation, every pair was able to conduct a continuous conversation in our experiment. We listened to partial recorded files during organization, and the content usually were ice-breaking topics related to college student life, such as major, grade, course, life in dormitory, student associations, and etc.

## Discussion

In this study, we found two factors that predispose individuals to joint movement with partners (i.e., motor synchrony) in a naturally social situation: (a) female pairs exhibited greater motor synchrony than male pairs; and (b) pairs with lower degrees of autistic traits synchronized better than those with higher degrees.

Interestingly, we found that females, in spite of their lower levels of physical activity[29,30] and worse motor performance than males[23–28], exhibited greater implicit motor synchrony in our experiment. Compared to other motion tasks, walking is almost effortless and automatic for all participants of both genders. Therefore, we speculate that motor skills are not the main force driving the gender difference here. Instead, additional social-cognitive factors in which females are known to be superior (e.g., empathy and social communication) might explain this advantage in walking synchrony. It is well known that females present higher levels of empathy than males, and empathy plays an important role in understanding other people’s intention, which is also crucial for social motor coordination[67,68]. And motor coordination tasks also strengthen empathy[68,69]. Together with previous study showing gender difference in non-verbal communication, it is likely that gender differences in motor coordination may be a characteristic of distinct social cognition and communication styles between females and males. Females’ superiority in non-verbal communication reflect from decoding non-verbal cues[36–44], emotional expression[41,45–47], and emotion regulation[33,48]. Our study further shows that females are also more responsive than men to other’s body movements, especially in terms of synchronizing their bodies to others’.

This study provides evidence showing that high autistic tendencies impede interpersonal motor synchronization in normal population, however the underlying mechanism needs further exploration. It’s noteworthy that a study with a large sample (N = 243) reported that autistic traits failed to predict automatic hand gesture imitation[70]. This may indicate difference between non-conscious mimicry and implicit motor synchrony, which remains a topic for further investigation. One may feel the separation investigation of “autistic trait” and “gender” redundant because statistics show that more males than females are diagnosed with autism[63,71,72]. In this study, our male participants possess significantly higher autistic traits (measured by AQ) than female participants. However, it is noteworthy that the result of gender difference on synchronization was based on ANCOVA, where we controlled AQ over the gender difference. This means that the gender difference we found was free from AQ score. In addition, the gender difference in autistic trait among non-clinical population is less conclusive. Except the first study that reported significant lower AQ in healthy male participants than female participants[63], subsequent studies showed no statistical difference between healthy males and females[73–78]. The only exception study that reported higher AQ in female population[79] was based on a much bigger sample size (723 male and 1038 female), and authors reported that the effect size was small (partial eta squared (η^2^) values was 0.03). Therefore, although ASD seems to be a gender defiant related disorder, the AQ score distribution between two genders among TD adults is not well established yet. From available research, gender and AQ in healthy population remain two factors worth separate investigation.

We observed a weak positive association between walking synchrony and interpersonal impressions. The demonstrated association was weak, possibly because of the implicit nature of the walking task ensured by a delicately designed cover story, while in other studies participants were explicitly instructed or cued to intentionally sync or not sync with partners and fully aware that the motor synchrony was being observed[34,80–82]. Explicit synchrony could boost perceptions of similarity[34,82], which was a known predictor for attraction[83]. In current study, implicit synchrony might be too subtle to create perceived similarity, which led to a weak effect on affection. Moreover, observers perceive, memorize, process information, and form decisions differently in conscious and unconscious states[84]. The effects of implicit and explicit movements in joint action warrant further investigation. The weak association may also be due to the in-group atmosphere in our test. Kato et al.[65] reported that in-group participants who conversed for an hour, unlike out-group participants, showed no correspondence between enhanced affection and footstep synchronization. Our design did not emphasize out-group labeling, which may attenuate the bodily synchrony effect on affection.

Our findings, along with several others, converged to a functional perspective that interpersonal motor synchronization is a form of social communication. Motor synchrony promotes emotion inference[85], empathy[68,69] and theory of mind[86]. It also cultivates positive impression[34,80–82], cooperation[87], affection[12], trust[81], and pro-social behavior in both adults[88,89] and infants[90,91]. Conversely, social factors related to others such as social attitude and competence[6,92], partners’ character[93], the psychosocial difference between two agents[9,10], and the level of two agents’ agreement[11,12] all modulate motor synchrony. We suggest that motor synchronization between two partners may carry social information for exchange and promotes social consequences, similar to other forms of non-verbal communication such as facial expressions, postures, and gestures that we use to express emotion, convey intention, and establish impression. This view is supported by electrophysiological studies which showed that implicit motor synchrony involves the social brain[94,95] (e.g., frontoparietal and centroparietal networks), which is also crucial for other social functions such as empathy[67,68]. Along this line of thinking, the gender difference in motor synchrony may simply reflect different communication styles between gender groups. Females are better at applying interpersonal motor synchrony than males, similar to how females excel in applying other nonverbal signals such as facial expressions[41,45,46], voice[47], and infant emotional self-regulation[33,48].

Our walking paradigm has the following advantages compared to other joint tasks. Firstly, we minimized observers’ awareness of the joint motion and offered a novel way to quantify the communication effects of implicit joint motion. We can further examine whether the characteristics of implicit communication resemble other forms of explicit joint actions observed in rituals (e.g. dancing and marching). Secondly, our paradigm ensured ecological validity, which has received increasing emphasis for both behavioral [96] and neural [97] studies on interpersonal interaction. We simulated a natural scenario of real social interaction, in contrast to past studies that involved participants performing repetitive movements jointly with partners, which is not common in daily life [2,3,93,98]. Moreover, evidence has shown that kinematics of actions vary across social contexts[96] and are modulated by social factors such as social intention[99,100] and group membership[101]. Therefore, implicit body coordination might function differently between real social interaction and repetitive action task in laboratory.

There are several directions for future research. Firstly, it will be desirable to have participants’ athletic and motor skills and levels of physical activity into consideration. Although walking is a daily activity for almost everyone, and all our participants seemed equally competent and comfortable in this task, additional motor skills measurements can offer information about individual differences. This is particularly useful for comparison across different gender or age groups with distinct physical activity levels[29,30] and motor abilities differences[23–28]. Secondly, the conversation effect on motor synchrony between a pair could be further included in the complete understanding of this form of implicit communication. Some studies explore the effect in terms of conversation content but the results were not consistent. Paxton and Dale[11] discovered that arguments inhibited the interpersonal convergence of body movements compared to less competitive conversations, while Tschacher et al.[12] found that a debate promoted greater levels of body synchrony than a cooperative conversation. One reason of the inconsistency might be that the degree of interests towards conversation topics varies among participants depending on the prior knowledge about the topics or preference. In such cases the involvement of conversation between pairs varies, which might also affect the motor synchrony. The involvement has been measured in terms of utterance overlap[102]. An endeavor for the control of conversation content as well as involvement of conversation might be required for further investigation for our complete understanding of this form of joint movement.

In sum, our study revealed that permanent traits predispose individuals to implicit bodily synchrony. Specifically, females synced better than males, and high autistic traits hindered interpersonal motor synchrony. Moreover, we provided evidence that implicit walking synchrony was positively associated with affection in an environment simulating a daily encounter.

## Supporting information

S1 File Autism-spectrum quotient (AQ) questionnaire (Chinese translation). (PDF)

S2 File Interpersonal judgment scale (IJS) (Chinese translation). (PDF)
