# Supplementary material for: Gender and autistic traits modulate implicit motor synchrony
Source: PLoS One. 2017 Sep 5;12(9):e0184083. doi: 10.1371/journal.pone.0184083 (PMC5584984; doi:10.1371/journal.pone.0184083)
Supplement: S1 File — (PDF) [file pone.0184083.s001.pdf]

Supporting Information I:

This is the interpersonal judgment scale (IJS) used in the study (translated to Chinese).
The original IJS questionnaire can be found:
IJS: in Vol. 11 of the book *The attraction paradigm*, written by Byrne, D.

請選擇最合適的一項來描述你的同行夥伴。

	強烈同意	同意	略為同意	中立	略為不同意	不同意	強烈不同意
此人的智力遠高於常人。							
此人的道德水平極高。							
此人對於人或環境難以適應。							
我很喜歡此人。							
我很討厭與此人一起工作。							
