# Supplementary material for: Gender and autistic traits modulate implicit motor synchrony
Source: PLoS One. 2017 Sep 5;12(9):e0184083. doi: 10.1371/journal.pone.0184083 (PMC5584984; doi:10.1371/journal.pone.0184083)
Supplement: S2 File — (PDF) [file pone.0184083.s002.pdf]

Supporting Information II:

This is the autism-spectrum quotient (AQ) questionnaire used in the study (translated to Chinese). The original AQ questionnaire can be found: https://link.springer.com/article/10.1023%2FA%3A1005653411471?LI=true

這份問卷將反映出您的溝通模式。請認真回答每一題，評估您對題中描述的同意程度，並圈出最合適的答案。

	完全同意	少許同意	少許不同意	完全不同意
例子：我願意冒險。		✓		
1. 我喜歡和別人一起做事多於單獨處事。				
2. 我較喜歡以相同方式重複做事。				
3. 如要想像事情，我覺得在腦海中構想該事情的畫面是容易的。				
4. 我常常太沉醉於某事情而忽略了其他東西。				
5. 我常常留意到一些別人不為意的細微聲音。				
6. 我常常留意車牌號碼或類似的數列資料。				
7. 即使我認為自己的說話很有禮貌，但別人常常告訴我那是很無禮的。				
8. 在閱讀故事時，我能輕易地想像出角色的模樣。				
9. 我對日期很著迷。				
10. 在社交場合中，我能輕易聽懂不同人的對話。				
11. 我覺得身處社交場合是輕鬆自在的事。				
12. 我傾向留意一些別人不為意的細節。				
13. 我喜歡去圖書館多於宴會。				
14. 我覺得虛構故事是容易的。				
15. 相對於物件，我對人有更大興趣。				
16. 我容易有強烈的喜好；如不能從事這些喜好，我會感到不開心。				
17. 我喜愛與別人閒談。				
18. 我說話時別人不容易插嘴。				
19. 我對數字很著迷。				
20. 在閱讀故事時，我覺得很難明白故事人物的意圖。				
21. 我並不特別喜歡閱讀小說。				
22. 我覺得很難結識新朋友。				
23. 我常常留意事物的規律模式。				
24. 我喜歡去戲院多於博物館。				
25. 當日常生活被擾亂，我不會感到煩躁。				

接下頁...

... 接上頁

	完全同意	少許同意	少許不同意	完全不同意
例子：我願意冒險。		✓		
26. 我不懂得怎樣持續一段對話。				
27. 我覺得理解對方說話的背後含意是容易的。				
28. 我通常留意事情的整體多於其細節。				
29. 我不善於牢記電話號碼。				
30. 我很少留意身處境地或別人外表的細微轉變。				
31.我會知道別人何時對我的說話感厭煩。				
32. 我覺得同時處理不同的事情是容易的。				
33. 在電話對談中，我不肯定何時應該發言。				
34. 我喜愛自發地做事。				
35. 我常常是最後一個才明白笑話意思的人。				
36. 我覺得從察看別人表情去了解他們的想法或感受是容易的。				
37. 若正進行的活動被打斷，我可以很快恢復進行該事情。				
38. 我善於與別人閒談。				
39. 別人常常告知我，我不斷重複做或說同一件事情。				
40. 當我較年幼時，我喜歡和小朋友玩假裝遊戲。				
41. 我喜歡收集某個種類(如車類、雀鳥類、火車類、植物類等)的資料。				
42. 我很難想像自己成為別人會是怎樣的。				
43. 我喜歡仔細計劃自己參與的每一項活動。				
44. 我喜歡社交場合。				
45. 我覺得理解別人意圖是困難的。				
46. 新環境令我緊張。				
47. 我喜歡認識新朋友。				
48. 我是個善於交際的人。				
49. 我不善於牢記別人的出生日期。				
50. 我覺得和小朋友玩假裝遊戲是容易的。				

感謝您的參與！
